# Retraction: Predicting the Herd Immunity Threshold during an Outbreak: A Recursive Approach

**DOI:** 10.1371/annotation/d78ba1b7-79d3-44f0-b632-224fb9149a23

**Published:** 2012-10-09

**Authors:** Nathan T. Georgette

The author wishes to retract this publication due to a mathematical flaw that undermines the article’s methods and conclusions.

This paper builds upon the author’s previous publication in Internet J Epidemiology [1]. In that previous paper, the author derived a mathematical model for the basic reproduction number from a modified SIR system of differential equations. During this derivation, an integration step was performed that implicitly assumed the per-susceptible-person rate of immunization, ρ, is constant for a given epidemic. At the time, the author did not recognize the existence of this assumption and unknowingly violated it while building upon [1] to develop this current paper: the author founded this work on a description of ρ as changing through time. This violation of an underlying assumption renders invalid the results of the current paper. 

The author apologizes to the readers and editors of PLOS ONE for this error and the delay in its recognition.

[1] Georgette N (2007) The Quantification Of The Effects Of Changes In Population Parameters On The Herd Immunity Threshold. Internet J Epidemiology 5(1).

